# Brain Synchrony in Competition and Collaboration During Multiuser Neurofeedback-Based Gaming

**DOI:** 10.3389/fnrgo.2021.749009

**Published:** 2021-11-01

**Authors:** Ioana Susnoschi Luca, Finda Dwi Putri, Hao Ding, Aleksandra Vuckovič

**Affiliations:** ^1^Biomedical Research Division, School of Engineering, University of Glasgow, Glasgow, United Kingdom; ^2^Section of Movement Disorders and Neurostimulation, Biomedical Statistics and Multimodal Signal Processing Unit, Department of Neurology, Focus Program Translational Neuroscience (FTN), University Medical Center of the Johannes Gutenberg-University Mainz, Mainz, Germany

**Keywords:** BCI, hyperscanning, functional connectivity, phase locking value (PLV), EEG, neurofeedback, gaming

## Abstract

EEG hyperscanning during multiuser gaming offers opportunities to study brain characteristics of social interaction under various paradigms. In this study, we aimed to characterize neural signatures and phase-based functional connectivity patterns of gaming strategies during collaborative and competitive alpha neurofeedback games. Twenty pairs of participants with no close relationship took part in three sessions of collaborative or competitive multiuser neurofeedback (NF), with identical graphical user interface, using Relative Alpha (RA) power as a control signal. Collaborating dyads had to keep their RA within 5% of each other for the team to be awarded a point, while members of competitive dyads scored points if their RA was 10% above their opponent's. Interbrain synchrony existed only during gaming but not during baseline in either collaborative or competitive gaming. Spectral analysis and interbrain connectivity showed that in collaborative gaming, players with higher resting state alpha content were more active in regulating their RA to match those of their partner. Moreover, interconnectivity was the strongest between homologous brain structures of the dyad in theta and alpha bands, indicating a similar degree of planning and social exchange. Competitive gaming emphasized the difference between participants who were able to relax and, in this way, maintain RA, and those who had an unsuccessful approach. Analysis of interbrain connections shows engagement of frontal areas in losers, but not in winners, indicating the formers' attempt to mentalise and apply strategies that might be suitable for conventional gaming, but inappropriate for the alpha neurofeedback-based game. We show that in gaming based on multiplayer non-verbalized NF, the winning strategy is dependent on the rules of the game and on the behavior of the opponent. Mental strategies that characterize successful gaming in the physical world might not be adequate for NF-based gaming.

## Introduction

Humans are social creatures whose behavior and consciousness are heavily shaped by their environment. Hence, it is natural that hyperscanning, a technique which involves simultaneous recording of physiological activity from more than one subject, is used to deepen our understanding of human interaction. In recent years, hyperscanning has been applied to brain activity to shed light on the neurophysiological representation of various types of interpersonal communication. These range from verbal interaction (Pérez et al., 2017; Ahn et al., 2018), leader-imitator (Dumas et al., 2010; Yun et al., 2012), joint attention and joint decision-making (Toppi et al., 2016; Hu et al., 2018), to teaching or playing music in a duet (Sänger et al., 2012; Müller et al., 2013). Moreover, the neurological coupling of mothers and their infants was investigated for positive and negative emotions and their regulation (Reindl et al., 2018; Santamaria et al., 2020). Beyond understanding the effect of social interaction on the brain, hyperscanning methods applied to larger groups (e.g., students in a classroom) facilitates research into effective communication and teaching methods (Dikker et al., 2017; Pan et al., 2020; Reinero et al., 2021). In the near future, the technology might be used as a training tool, to promote affectivity and social awareness (Järvelä et al., 2021), or to improve the process of group decision-making (Bhattacharyya et al., 2019).

Trademark interactions between humans include working together toward a joint goal (collaboration), or working individually, seeking to assert dominance (competition). Two brains working toward a joint goal have been found to be more functionally interconnected than two brains competing with each other (Balconi and Vanutelli, 2018), but the latter case demands a greater ability to mentalise, or represent others' thoughts in one's brain (Decety et al., 2004).

Functional integration of brain regions is highly dependent on the type of task. Synchronized motion in a dyad leads to synchrony of brain processes localized to central and posterior regions (Dumas et al., 2010). Hypersynchrony as a result of cooperation was found in the frontocentral theta, frontal and temporal beta and pre-frontal and centroparietal alpha band (Dumas et al., 2010; Yun et al., 2012; Toppi et al., 2016; Pérez et al., 2017; Hu et al., 2018; Cho et al., 2020), and in high and low beta bands (Müller et al., 2013). Research literature proposes that interbrain synchrony is either a discriminator for, or a result of behavioral synchrony (Dumas et al., 2010; Yun et al., 2012), induced by decision-making (Hu et al., 2018), and turn taking speaking or playing an instrument (Müller et al., 2013; Pérez et al., 2017).

There is less of an agreement in the case of competing dyads, where studies reported both increase in low beta phase locking due to the interaction (Cho et al., 2020), and no significant change from baseline (Balconi and Vanutelli, 2018), but a decrease in frontal alpha interbrain coupling (Balconi and Vanutelli, 2018). Studies directly comparing competitive and collaborative interaction found differences in beta band hyperconnections (Sinha et al., 2017; Cho et al., 2020). When both members of a dyad decide to defect rather than cooperate or retaliate, their frontal interconnectivity decreases (Babiloni et al., 2007; De Vico Fallani et al., 2010). In addition, recent findings suggest that interbrain connectivity does not necessarily facilitate social interaction (Mayo and Gordon, 2020), and that neural and behavioral synchrony might complement each other in dyads with close, well-established relationships (e.g., couples), (Djalovski et al., 2021).

Social interaction occurs in two dimensions: as exchange of information between actors, and as metalized or perceived interplay by each of them. Therefore, to obtain a complete picture of the interaction, it is necessary to look at both intrabrain and interbrain changes in connectivity.

Passive applications of multibrain brain-computer interface (BCI) games are used to assess players' mental state and modify the game in real time to improve experience (Stevens et al., 2010; Darzi and Novak, 2021), and hybrid gaming combines neural activity (motor imagery, somatosensory evoked potential) with gestures and speech (Krepki et al., 2007; Bonnet et al., 2013). Studies involving active multiplayer BCI-based gaming such as Brain Ball or Brain Arena report on team/user experience (Bonnet et al., 2013; Gürkök et al., 2013). BCI hyperscanning techniques provide a large ground for diverse research involving different sensing techniques, paradigms, and player interactions.

Neurofeedback (NF) is a closed-loop version of active BCI whereby the subject attempts to modulate their brain activity to meet a certain target (e.g., surpass a pre-defined threshold). NF-based BCI gaming differs from motor imagery or evoked potential paradigms in that the former uses a form of learning (operant conditioning), while the latter relies on the participant receiving specific instructions, e.g., *imagine moving your wrist*. Learning how to change one's brain activity has a cumulative effect over training sessions (Ros and and Gruzelier, 2011), and an instantaneous effect on neural processes, as demonstrated with functional connectivity analysis (Ibric et al., 2009; Imperatori et al., 2017).

We aimed to learn more about the neural substrates of non-verbal social interaction during neurofeedback-based operant conditioning, and how these are shaped by competition and collaboration.

In the present study, NF-based two-user gaming was employed to investigate the effects of collaborating and competing on the dyad's neural mechanisms. Thus, the players' task was two-fold: to voluntary modulate their brain activity to accomplish the game's target, and to interact with each other, either cooperating or competing. Phase locking value was applied to quantify inter and intrabrain synchrony. We addressed the following research questions:

1. How does indirect, non-muscular social interaction during neurofeedback affect players' oscillatory brain activity?
2. Are there any differences in neurofeedback approaches between competing players in competitive gaming?
3. Are there any differences in neurofeedback approaches between collaborating players in collaborative gaming?
4. What are the characteristics of interbrain wave synchrony in dyads in two different gaming paradigms?

## Methods

### Experimental Paradigms

**Figure 1:**
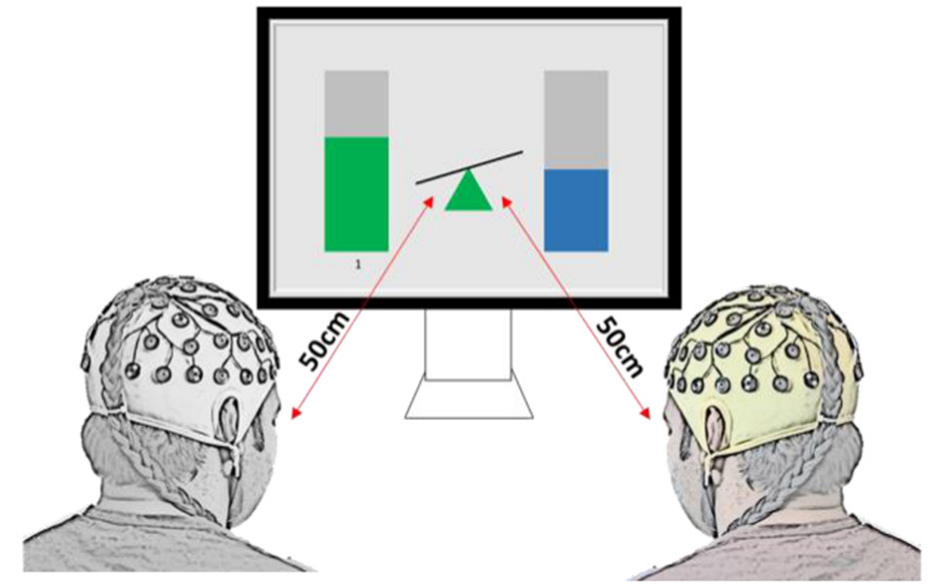
Experimental setup and graphical user interface for the two-player BCI game, for both collaborative and competitive paradigms. In collaborative game, the task was to keep the seesaw in the middle in balance, while in the competitive game the task was to tilt it toward the player (as if being heavier in the physical world).

Ten pairs (twenty healthy able-bodied adults, age 26.9 ± 3.7 years old, eight females and twelve males) participated in the collaborative gaming experiments and ten pairs (twenty able-bodied adults, age 26.9 ± 4.7 years old, eight females and twelve males) took part in the competitive gaming experiments. None of the participants took part in both paradigms. Dyad members were not in a relationship or close friends, although some knew each other prior to taking part in the experiment. Pairs were sat next to each other, facing a computer screen, as shown in Figure 1. They were instructed not to speak or move during the game. An experimenter (outside of participants' visual filed) observed all gaming sessions to make sure that participants complied with the instructions. To exclude the influence of variation of a visual feedback, the Graphical User Interface (GUI) was identical in both gaming paradigms.

Each pair participated in three (collaborative or competitive) gaming sessions with identical experimental protocol. Each session had the following structure: 2 minutes eyes open (EO) resting state EEG recording, 2 minutes eyes closed (EC) EEG recording, 30 minutes of gaming (six sets of 5 minutes), followed by 2 minutes EO EEG. Since both the collaborative and the competitive tasks were based on neurofeedback, a non-verbalized operant-conditioning method which requires practice (Gruzelier, 2014; Hassan et al., 2015), sessions 1 and 2 were used as NF training, and electroencephalogram (EEG) was recorded only at Pz. Session 3 EEG was recorded from 16 locations, following 10–10 electrode placement (AF3, AF4, FC3, FC4, C3, Cz, C4, P3, Pz, P4, O1, Oz, O2). All analysis was performed on data from session 3.

EEG was recorded at a sampling rate of 256 Hz using g.USBamp biosignal amplifiers (Guger Technologies, Austria). During the game, data was analyzed in real time in Matlab (R2015a, The Math works, Inc., USA) and Simulink, and presented to the participants on a GUI. The signal was band-passed in the alpha (8–12 Hz) and wider band (2–30 Hz) ranges using a 5th order infinite impulse response (IIR) digital Butterworth filter (g.USBamp biosignal processing blocks, Simulink) over 0.5 s moving average windows updated every 8 samples. Relative alpha power (RA) was computed as the power in the band of interest divided by power in the wider 2–30 Hz band (Eq. 1). The RA at Pz was calculated in Simulink and sent as a control signal to the GUI.


(1) $$\begin{array}{r} RA\left(\%\right)=\frac{P_{alpha}}{P_{2\;-\;30}}\cdot 100 \end{array}$$

The brain computer interface was the same for collaborative and competitive gaming, but the aim differed. Modulation of relative alpha (RA) was shown in real time, as bars on either side of a seesaw (Figure 1). Increasing or decreasing RA at Pz led to a proportional change in bar height, and controlled the incline of the seesaw. To account for the natural variation in people's resting-state alpha power, the player with higher resting state alpha power had their RA normalized during gaming by a factor equal to the ratio between lower RA and higher RA (Normalizing coefficient, NC, Equation 2).


(2) $$\begin{array}{r} \text{NC}=\frac{RA_{low}}{RA_{high}} \end{array}$$

#### Collaborative Task

Pairs who played in collaborative mode were required to maintain their RA within 5% of each other, thus keeping the seesaw in balance. A point was awarded for every 0.5 s they kept the seesaw balanced, and the score was displayed on the screen. For the purpose of the analysis, collaborative players were grouped in dominant (D) and non-dominant (ND), where D had the higher resting state (EO baseline) RA at Pz before the start of session 3.

#### Competitive Task

In competitive mode, the aim was for players to increase their RA to 10% above that of the competitor's and incline the seesaw to their side. Doing so for 1 second awarded them one point. The score was displayed on the screen. After data collection, competitive gaming players were grouped into winners (W) and losers (L), based on their score in session 3.

### Offline EEG Pre-processing

The raw EEG signal was band-pass filtered in the 2–30 Hz band using a 5th order IIR digital Butterworth filter. For session 3 data (multi-channel), pre-processing was performed using Infomax independent component analysis (ICA) implemented in EEGLAB v14.1.2b (Delorme and Makeig, 2004). Independent components containing eye blinking were removed (typically 1-2 components) and portions of signal with amplitude exceeding 100 μV present on most electrodes were manually removed.

### Connectivity

Connectivity was assessed by calculating the phase locking value (PLV) between sensors (intrabrain), or between sensors on two different brains (interbrain), on non-overlapping 0.5 s EEG epochs extracted from the onset of participants scoring a point. The number of trials for each participant or team was thus equal to the number of points scored. One pair was removed from collaborative analysis as their score was too low, therefore too few trials would have been used for analysis (excluded pair overall score = 40; mean ± std score = 6.7 ± 1.2, next lowest scoring pair overall score = 179; mean ± std score = 29.8 ± 7.0; Supplementary Figure S1). Baseline connectivity was determined from non-overlapping 0.5 s epochs extracted from 2 minutes of EO EEG recording for each participant.

It is hypothesized that two brain areas that are connected oscillate with similarly evolving instantaneous phases (Bruña et al., 2018). Intertrial PLV was computed on a 4 Hz window centered around the frequency of interest, *f*. The clean EEG signal was therefore bandpassed between [*f*−2*Hz*; *f*+2*Hz*] with a finite impulse response filter (Lachaux et al., 1999). The filter order was computed so as to include five cycles of the lowest frequency, *f*−2:


(3) $$\begin{array}{r} FIR\;order=5\frac{F_{s}}{f-2Hz} \end{array}$$

In this study, the Hilbert transform was employed to extract the instantaneous phase of the signal from bandpass filtered data (Tass et al., 1998). The frequency bands of interest, defined as cut-off frequencies, were theta (4–7 Hz), alpha (8–12 Hz), lower beta (13–20 Hz) and higher beta (21–30 Hz). The lower and higher beta bands were further divided into beta low 1 (13–16 Hz), beta low 2 (16–20 Hz), beta high 1 (21–25 Hz) and beta high 2 (26–30 Hz), and the corresponding filter order was computed from Eq. 3. Trial-averaged PLV, also called inter-site phase clustering, was computed as the average of phase angle differences between electrodes, over trials (Equation 4), (Cohen, 2014).


(4) $$PLV_{t}=\frac{1}{N}\left|\sum_{n=1}^{N}\text{exp}(j(\varphi_{1}\left(t,n\right)-\varphi_{2}(t,n))\right|$$

Where *N* is the total number of trials, φ_1_(*t, n*) and φ_2_(*t, n*) are the instantaneous phases of sources 1 and 2, at time *t*. PLV takes values between 0 and 1, with one being equivalent to complete synchrony (Lachaux et al., 1999).

PLV matrices were generated by averaging non-overlapping 0.5 s windows. PLV values for the four lower and higher beta bands were averaged respectively into beta 1 (13–20 Hz) and beta 2 (21–30 Hz) before performing the statistical analysis.

#### Intra-and Inter-brain Connectivity

PLV is a measure of functional connectivity based on phase synchrony between sensors, with no dependency on anatomical connectivity. Therefore, inter-brain and intra-brain PLV were extracted following the same methodology. PLV is non-directional (Cohen, 2014), and results are presented as non-directed topographical plots. To isolate the effects of gaming on functional integration of brain structures (Pérez et al., 2017), we subtracted resting state EO PLV from gaming PLV. Linear subtraction is possible because PLV is not affected by 1/f power scaling (Cohen, 2014). Therefore, the intrabrain results represent the change from baseline, due to the task.

### Spectral Analysis

Power spectrum density (PSD) was estimated on 0.5 s epochs extracted while the team/the player was scoring a point. The epochs were concatenated and Welch's periodogram (Signal Processing Toolbox, Matlab R2021a) was applied on 2 s segments with no overlap, windowed using the Hann function. The relative power content in the frequency bands of interest (theta, 4–7 Hz; alpha, 8–12 Hz; beta 1, 13–20 Hz and beta 2, 21–30 Hz) was computed with respect to the wide 2–30 Hz band. Spectral analysis was performed using the STUDY structure in EEGLAB (v.2021.0, Delorme and Makeig, 2004).

The PSD analysis was performed for both absolute alpha power and for relative alpha power (the percentage of alpha power within the overall 2–30 Hz EEG power). A difference between relative and absolute power is that relative power shows whether participants selectively upregulated alpha power, as compared to the power in other frequency bands. On the other hand, absolute power shows changes in EEG power in a selected frequency band between the baseline and NF irrespective of the changes in power in other frequency bands. Thus, a person can e.g., significantly increase the relative power without significantly changing the absolute power and vice versa. The former is of relevance for this particular study because of the rule of the game. The latter is more common in neurophysiology studies and is used by default in EEGLAB analysis.

### Statistical Validation

Hypothesis testing was performed using non-parametric permutation tests with a significance value set at *p* <0.01 for the connectivity analysis, and *p* <0.05 for the spectral analysis. Benjamini-Hochberg False Discovery Rate (FDR) correction for multiple comparisons was applied to the resulting *p*-values (Benjamini and Hochberg, 1995) from Matlab Multiple Testing Toolbox (Martínez-Cagigal, 2021). FDR controls for the proportion of type I errors (incorrect rejections of the null hypothesis). All analysis was performed in Matlab (The Math works, R2021a).

Phase locking value is considerably affected by volume conduction (intrabrain) and may indicate spurious connectivity (intra and interbrain), (Burgess, 2013). To account for this, the intra-and inter-brain connectivity was tested for significance against a null distribution constructed with surrogate data (Lachaux et al., 1999). The surrogate dataset was created for each participant, each electrode by shuffling the phases of the real signal and keeping the amplitude information, as described in Toppi et al. (2012). This method was repeated 1,000 times to create an empirical null dataset. PLV of the shuffled phase signal was computed for each repetition. The “true” grand average PLV was compared to the distribution of the 1,000 shuffled phase PLV values, for each electrode pair and each participant, and the significance level was set to 0.01. To determine if intrabrain connectivity was significantly different during gaming as compared to baseline, we used confidence interval bootstrapping (Statistics and Machine Learning Toolbox, Matlab R2021a) with 10,000 random draws. Only connections that passed the statistical validation test are presented in Results.

A permutation test with 10,000 repetitions was performed to determine whether the difference in relative power in all frequency bands between dominant and non-dominant (collaborative), or winners and losers (competitive) was significant, by mixing the players and computing the unpaired *t*-test statistic between fake D-ND or W-L pairs. Similarly, the relative power change between baseline and gaming was determined using 10,000 permutations of each group in the two conditions and performing a paired *t*-test (Delorme, 2006). A *p*-value lower than 0.05 was considered significant and FDR was applied to correct for multiple comparisons in all cases (*p*-value = 0.05).

#### Interbrain Connectivity

To test the validity of interbrain connectivity results, we performed a permutation test with 2,000 repetitions for the baseline and gaming cases, in addition to the aforementioned surrogate dataset comparison. Namely, we split all players of each team in two groups–Players 1 containing one player from each team, and Players 2–the remaining player in each team. We shuffled the labels of Players 1 and formed new (fake) teams before computing the interbrain phase locking value, and repeated this 2,000 times to construct the null distribution (Santamaria et al., 2020). As the number of trials varied between teams, the lowest number of trials of each two subjects was considered (Reindl et al., 2018). We then compared the real pairs' PLV value to this distribution using a Z-score. Thus, we tested whether a PLV value could arise from two players who are not in the same team and set the acceptance level at *p* = 0.01. Resulting *p*-values were corrected for multiple comparisons using FDR (*p*-value = 0.01).

A strength threshold was applied to interbrain connectivity data at 10% strongest connections to increase readability while preserving the patterns (Santamaria et al., 2020).

### Electrode Grouping

For visualization purposes, electrode locations were grouped into midline frontal (Fz), left frontal (AF3, F3, FC3), right frontal (AF4, F4, FC4), central (C3, Cz, C4), parietal (P3, Pz, P4) and occipital (O1, Oz, O2). The area grouping was decided upon after visually inspecting the plots, to ensure that connection patterns were maintained. Lateralization was only evident in the frontal area, therefore the central, parietal and occipital were grouped together, respectively.

### Questionnaires

To assess the mental workload of the participants in the two gaming paradigms, NASA Task Load Index (TLX) questionnaire was administered after each gaming session. The questionnaire addresses the mental demand, physical demand, temporal demand, performance, effort and frustration (Hart and Staveland, 1988).

## Results

In this section, results of the collaborative gaming are presented first, followed by results of the competitive gaming.

### Collaborative Gaming

**Figure 2:**
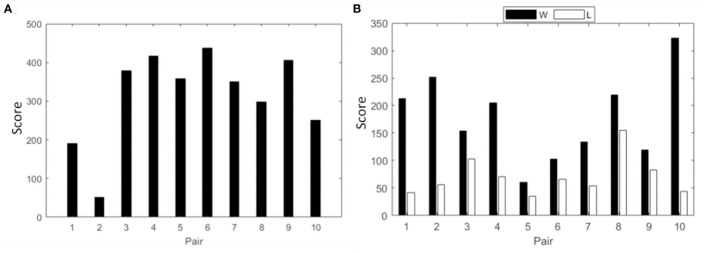
Cumulative score of all pairs from session three in **(A)** collaborative gaming; **(B)** competitive gaming, winners (black) and losers (white).

Spectral power analysis followed by the intra and interbrain analysis of PLV is presented for the collaborative gaming paradigm. Team scores for session 3 are shown in Figure 2A.

#### Spectral Analysis

**Figure 3:**
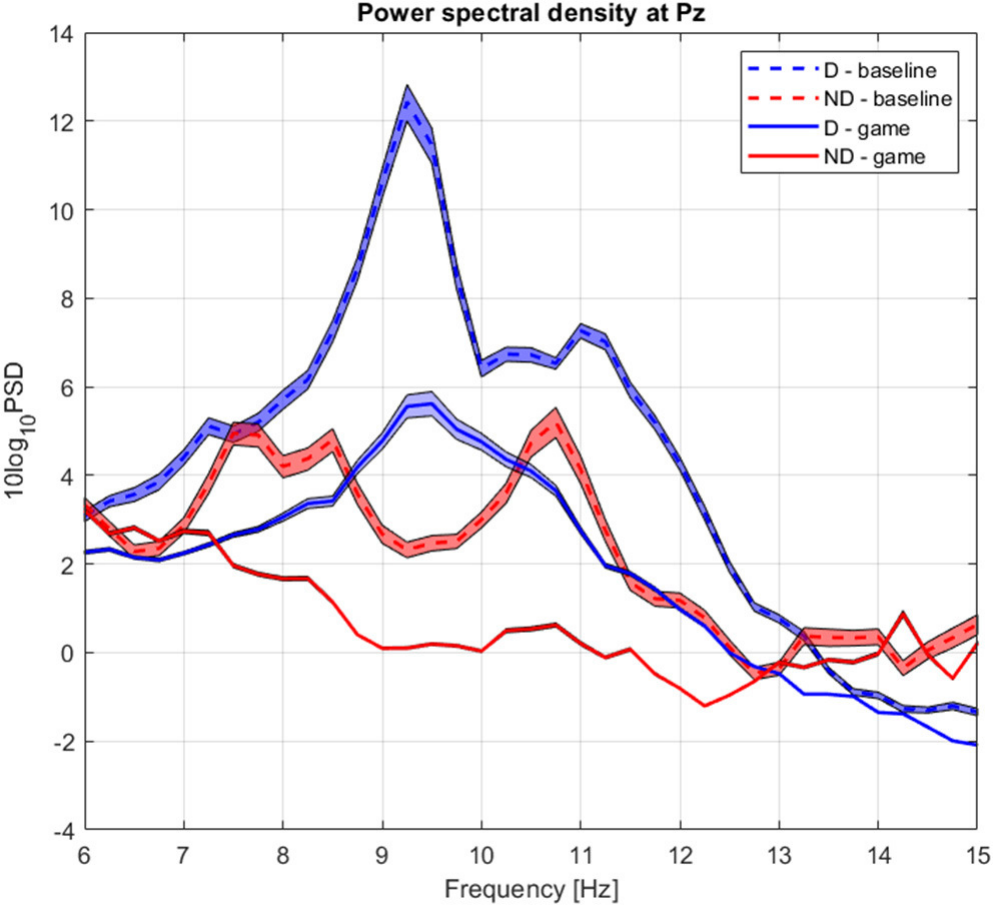
Group average power spectrum density during baseline (dashed) and gaming (continuous) for D (blue) and ND (green) players. Shaded area = standard deviation of the mean.

**Figure 4:**
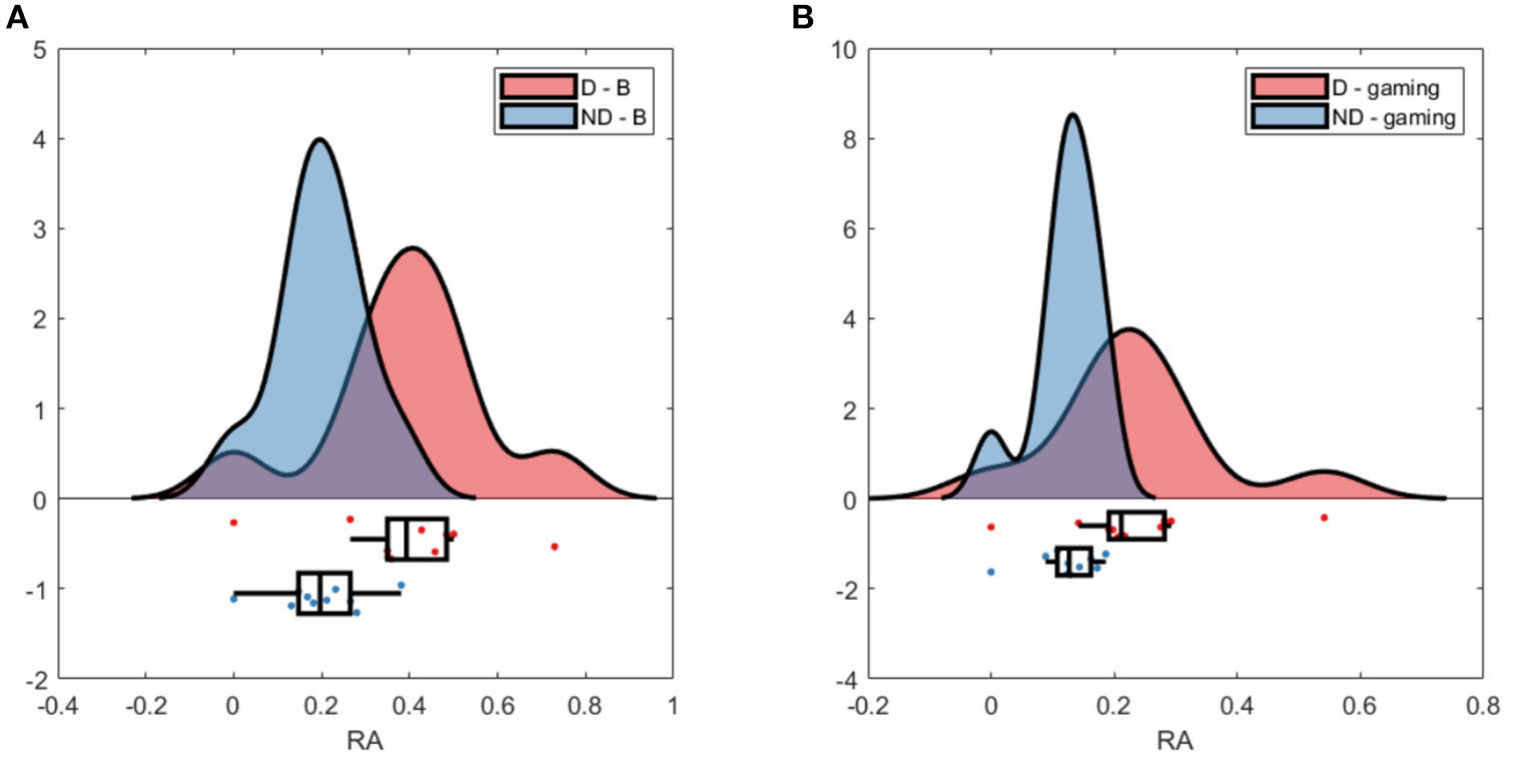
Raincloud plot of relative alpha power at Pz; **(A)** baseline D (red) and ND (blue); **(B)** gaming D and ND. Pair 2 excluded.

The group average PSD of the two groups during baseline and collaboration is presented in Figure 3. We analyzed changes in absolute power in the four frequency bands during baseline and NF (Figure 4), as well as the RA power at Pz, as this was the control signal for NF. Individual pairs' RA and topographical plots of RA can be found in the Supplementary Figures S2, S3, respectively.

The RA at Pz was significantly higher during baseline for D than for ND group (p_Pz_ <0.007), as well as during gaming (p_Pz_ = 0.009), not accounting for the normalizing coefficient used to balance D alpha during the experiment (Figure 4). Both groups significantly decreased the RA during gaming (at Pz p_D_ = 0.02, p_ND_ = 0.01).

**Figure 5:**
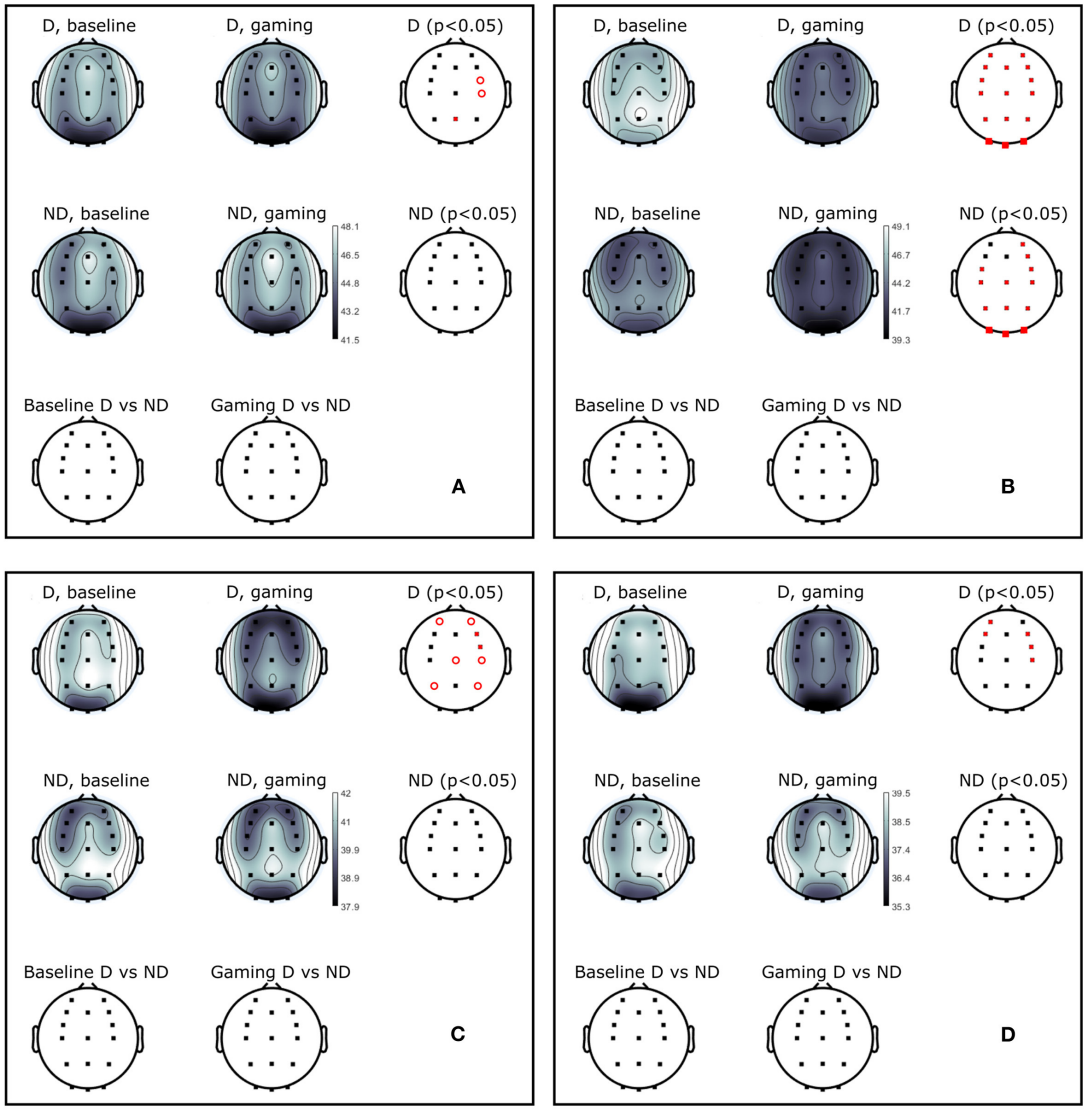
Power (dB) during baseline and collaborative gaming for D and ND groups, in **(A)** theta (4–7 Hz), **(B)** alpha (8–12 Hz), **(C)** beta 1 (13–20 Hz), and **(D)** beta 2 (21–30 Hz) bands. Statistical difference between groups (D, ND) and conditions (baseline, game) indicated in red (*p* <0.05, open circles–before; closed circles–after FDR correction).

The absolute alpha power at Pz was significantly higher during baseline in D as compared to ND group (p_Pz_ = 0.04), but they had comparable values during gaming (p_Pz_ = 0.2, Figure 5B). The absolute power significantly decreased during gaming at all electrode locations in D group (p_Pz_ = 2 ×10^−5^), and all but left frontal and anteriofrontal in ND group (p_Pz_ =1.6 ×10^−5^, Figure 5). Moreover, the dominant peak of the absolute alpha power at Pz in both groups decreased during gaming, as well as the overall alpha power. Contrary to what was expected, while players followed the rules of the game, they did not increase the RA, because that was not an explicit condition to score.

Theta power decreased in D players during gaming at Pz and right central and frontocentral electrodes. Power in beta 1 and beta 2 bands was significantly lower during gaming as compared to baseline at right frontal and central, and left anterio-frontal locations. Central and parietal decrease in beta 1 band did not remain significant after correction for multiple comparisons. Aside from significant changes in the alpha band, ND group did not modulate power in any other frequency bands.

#### Intra Brain PLV

**Figure 6:**
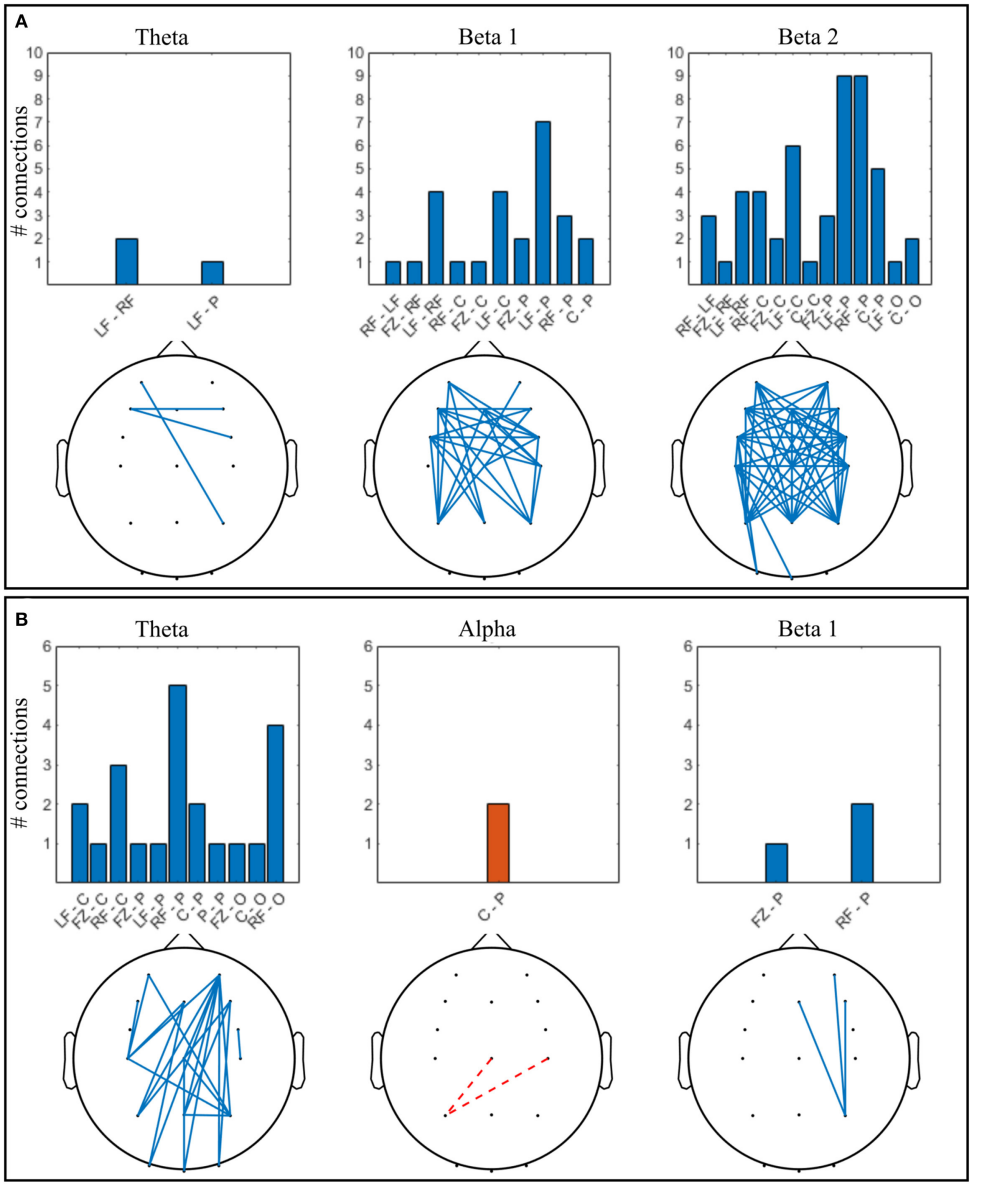
Significant connections in collaborative gaming vs. baseline grand average (*p* <0.01, FDR corrected); theta = 4–7 Hz, beta 1 = 13–20 Hz, beta 2 = 21–30 Hz. Panel **(A)** dominant (D) players; **(B)** non-dominant (ND) players. Grouped locations: LF, left frontal; RF, right frontal; C, central; P, parietal; O, occipital and topographical plot; Blue, continuous, increase from baseline; orange, dashed, decrease from baseline.

Results of two-tailed bootstrap testing and the number of significant connections between electrode groups (Section Electrode Grouping) are presented in Figures 6A,B for D and ND groups respectively, *p* <0.01 after FDR correction. In both groups, connectivity significantly increased in the theta and beta 1 bands. Alpha connectivity remained unchanged in D and decreased in ND group from baseline to gaming, while beta 2 connectivity densely increased in D but did not change in ND.

Increase in ND theta connectivity is mainly long-range, between the right frontal and the parietooccipital cortex, whereas frontal interhemispheric connections are present in the D group. Left anteriofrontal to right parietal locations are connected in both groups. No significant change was found in the alpha band between baseline and gaming in the D group (Figure 6A). In the ND group (Figure 6B), connectivity decreased in the alpha band between left parietal and central regions (*p* = 0.001). In D group, there is notable increase in connectivity in both beta 1 and beta 2 bands, frontally and centrally across hemispheres, and frontoparietally. This is not the case for ND group, where beta 1 connectivity increases only between the right parietal and midline-right anteriofrontal zones, and there is no significant change in beta 2 band.

#### Interbrain PLV

**Figure 7:**
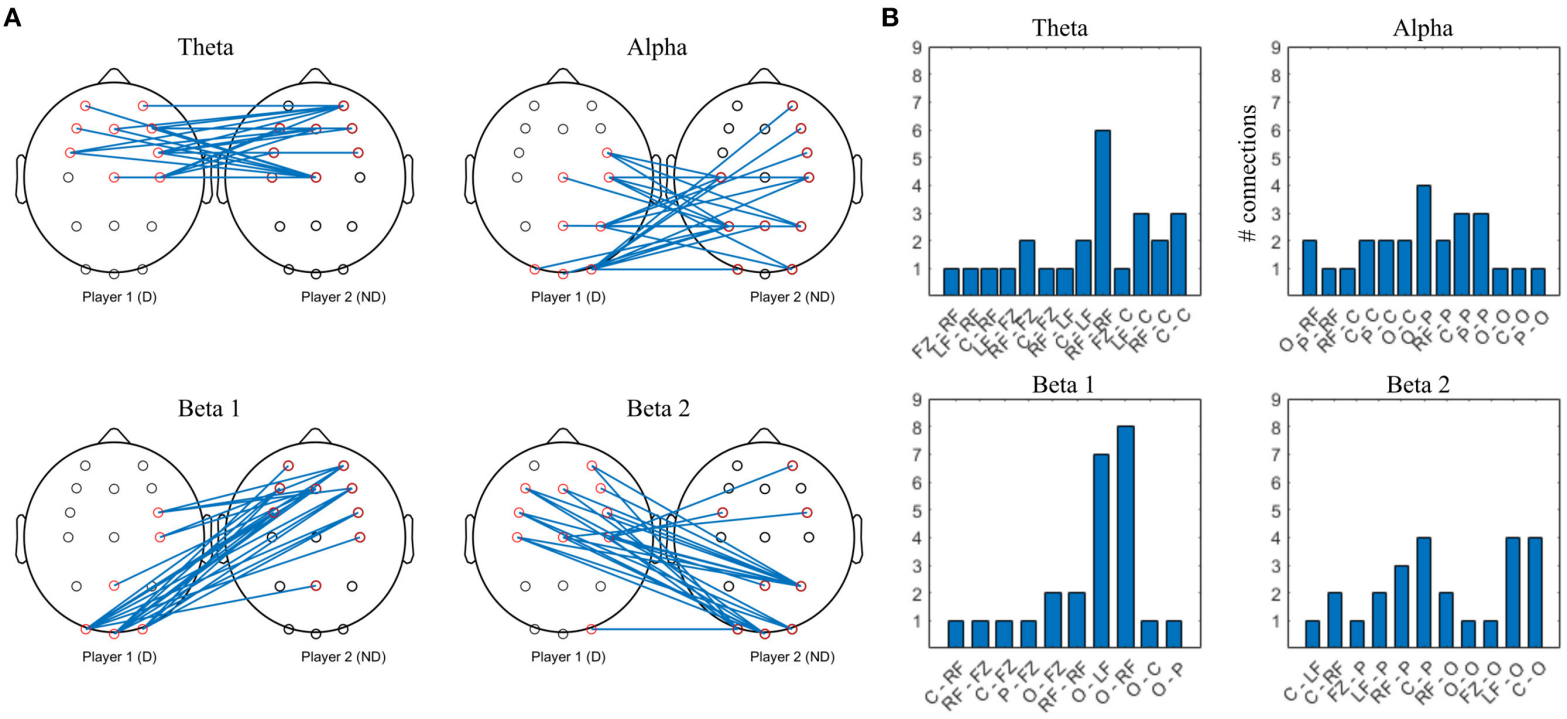
Top 10% strongest connections in collaborative interbrain connectivity; theta = 4–7 Hz, alpha = 8–12 Hz, beta 1 = 13–20 Hz, beta 2 = 21–30 Hz. **(A)** topographical plot. **(B)** grouped locations. Player 1 (D) first letter; Player 2 (ND) second letter. LF, left frontal; RF, right frontal; C, central; P, parietal; O, occipital.

After FDR correction, no baseline interbrain PLV passed the permutation test, leading to the conclusion that inter-brain PLV values in the resting state cannot be interpreted as true inter-brain connectivity. All gaming interbrain PLV values passed the statistical test, allowing interpretation of the PLV as resulting from interaction between players of the same team. Figure 7 shows the strongest 10% interbrain connections in all frequency bands. Violin plots showing individual pairs' interconnectivity values are presented in the Supplementary Figure S4.

The strongest theta interconnections occur between bilateral frontocentral locations of D and ND, seemingly symmetrical, with right frontal to right frontal synchrony dominating (Figure 7B). The strongest alpha interconnectivity arises between central and posterior cortex of D and ND, including Pz–Pz, and right frontal locations of ND. In D group, occipital locations are the most densely connected, followed by central and parietal (Figure 7B), whereas in ND, parietal dominate and there is more involvement of the left hemisphere. In beta 1 band, intersynchrony dominates between occipital locations of D and frontocentral ND, as well as right frontocentral (D) and right frontal (ND). Beta 2 intersynchrony is dominated by bilateral frontocentral (D)–parietooccipital (ND) cortices. Patterns of the strongest interconnections in the beta 1 and beta 2 bands appear to be mirrored: parieto-occipital locations of D are connected to frontal/frontocentral ND areas bilaterally in beta 1, while D frontal and left central are connected to ND parieto-occipital sites in beta 2.

### Competitive Gaming

In this section, spectral power analysis followed by the intra and interbrain analysis of PLV is presented for the competitive gaming paradigm. Scores of competitive gaming winners and losers are shown in Figure 2B.

#### Spectral Analysis

**Figure 8:**
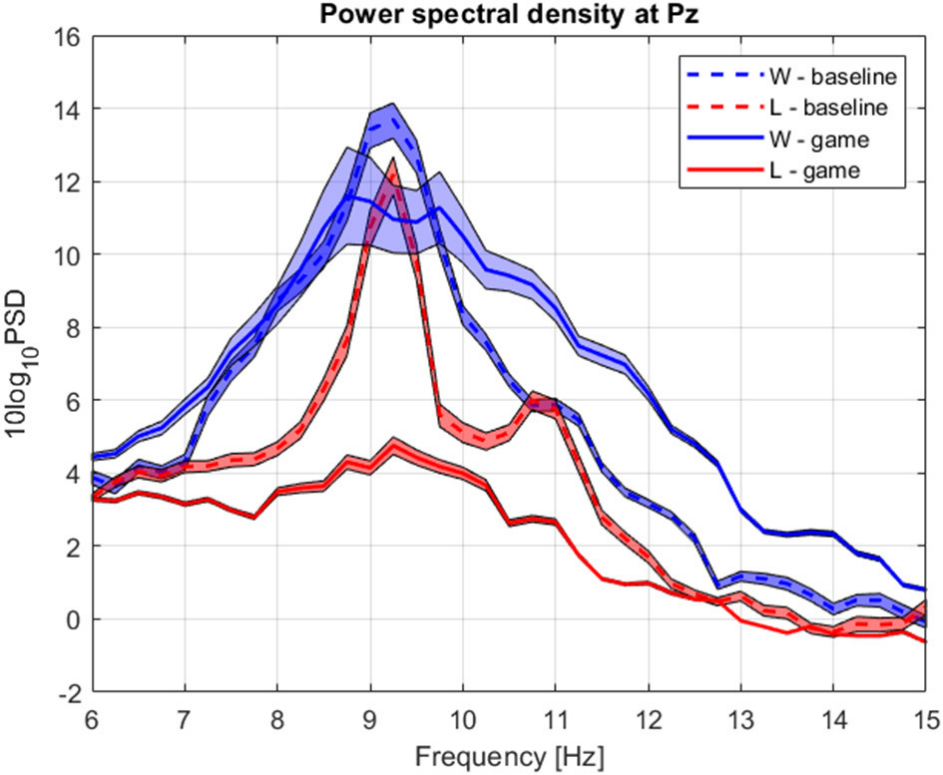
Group average power spectrum density during baseline (dashed) and competitive gaming (continuous) for winners (blue) and losers (red). Shaded area, standard deviation of the mean.

**Figure 9:**
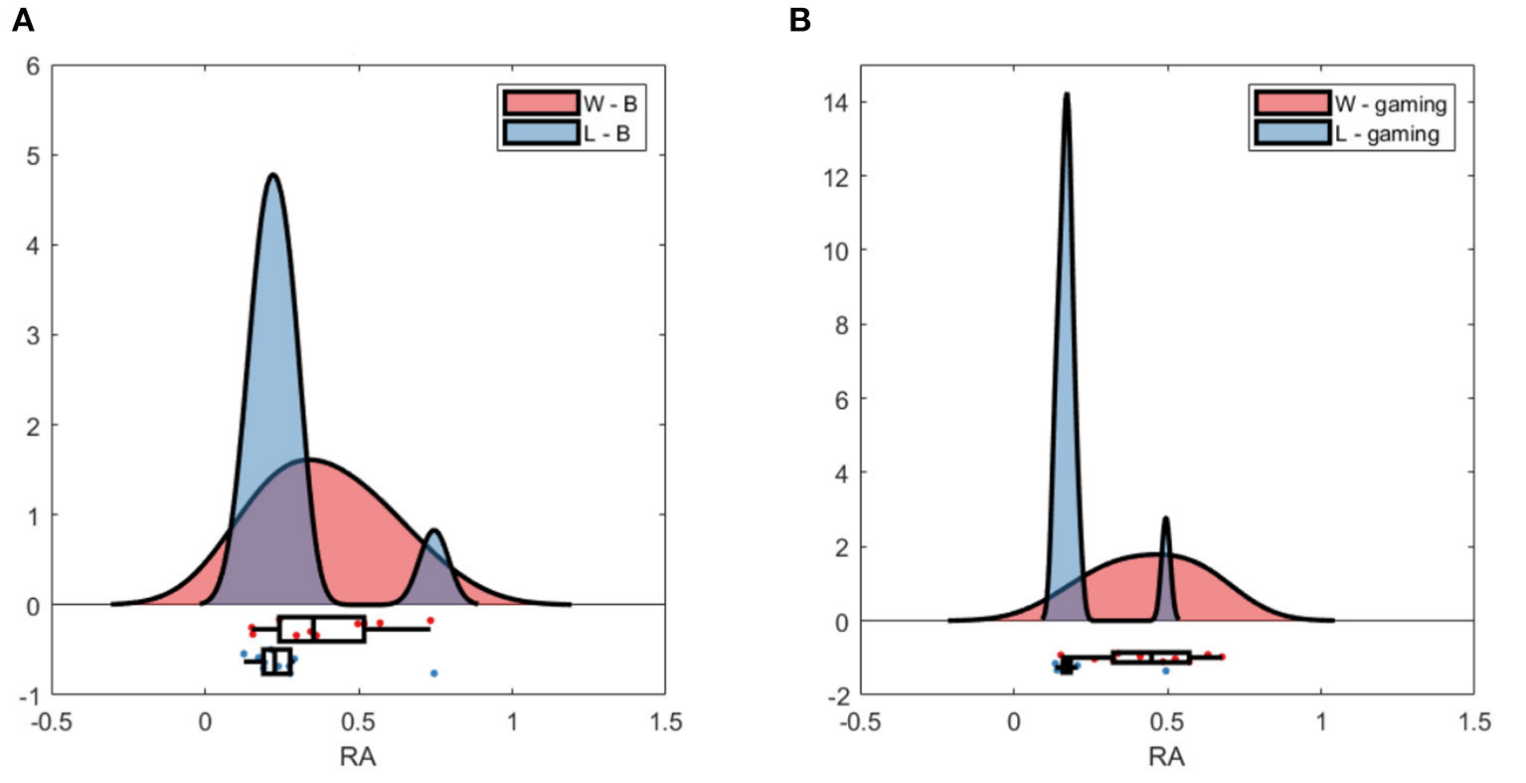
Raincloud plot of relative alpha power at Pz; **(A)** baseline W (red) and L (blue); **(B)** gaming W and L.

The group average PSD of the two groups during baseline and collaboration is presented in Figures 3, 8. Both groups decreased the amplitude of the dominant alpha peak during gaming. Individual pairs' RA during baseline and gaming are shown in Supplementary Figure S5. In contrast with collaborative gaming, where participants were grouped based on baseline RA, the difference in baseline RA of W and L (locations Pz and C4) did not remain significant after FDR correction (Figure 9 and Supplementary Figure S6). During gaming, W had a significantly, generated using (Allen et al., 2019), higher RA than L, at Pz (*p* = 0.019). However, comparison between baseline and gaming revealed that W did not change RA with respect to its baseline (p_Pz_ = 0.56), while L significantly decreased RA (p_Pz_ = 0.003).

**Figure 10:**
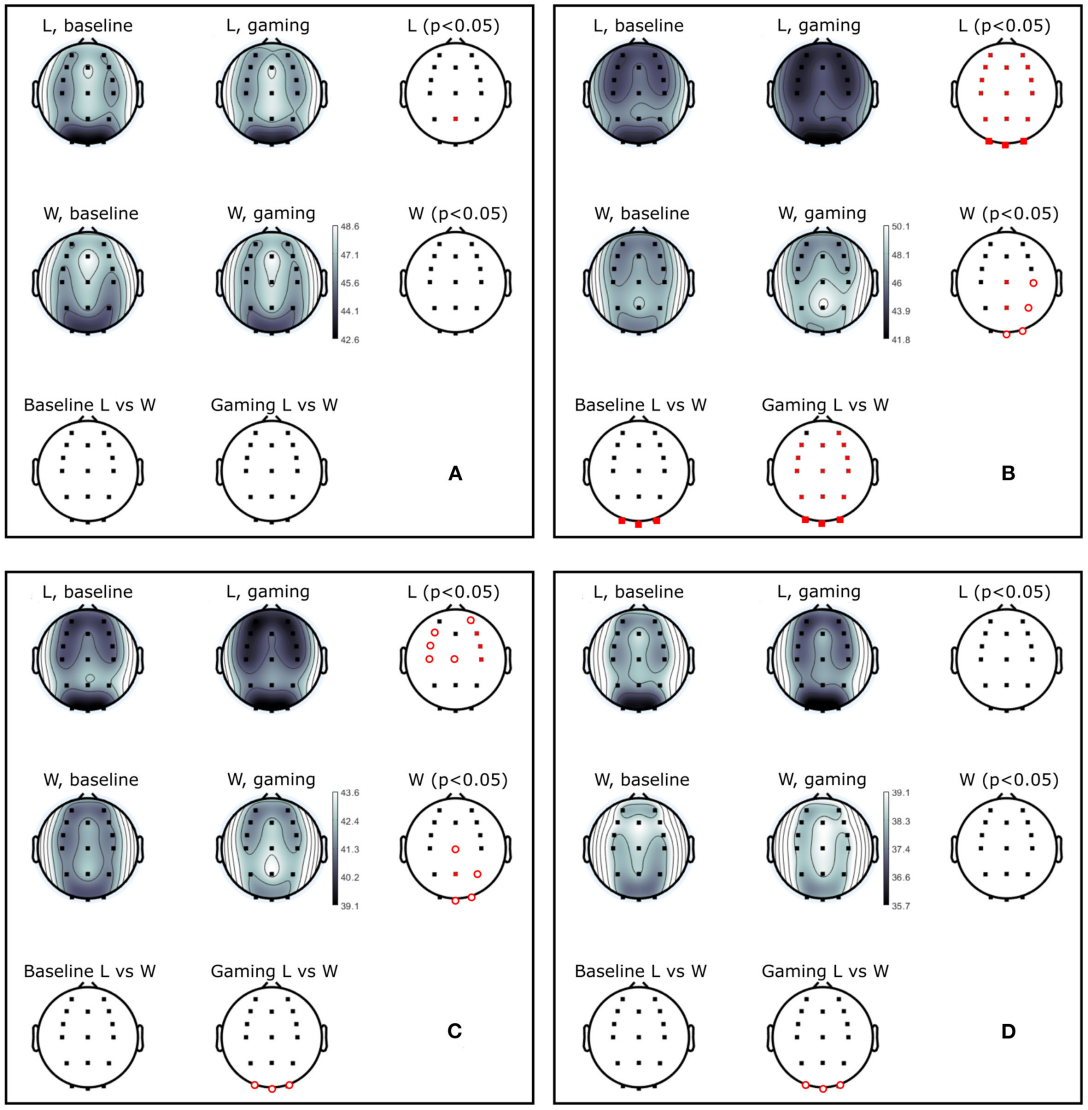
Power (dB) during baseline and competitive gaming for L and W groups, in **(A)** theta (4–7 Hz), **(B)** alpha (8–12 Hz), **(C)** beta 1 (13–20 Hz), and **(D)** beta 2 (21–30 Hz) bands. Significant differences from permutation testing shown in red (*p* <0.05, open circles–before; closed–after FDR correction).

Baseline absolute alpha power was significantly higher in W compared to L at occipital sites (Figure 10B) and gaming alpha was different at all locations, including Pz, between groups. The difference in gaming alpha was due to two factors: a significant decrease of alpha in L group (p_Pz_ = 0.008) and increase in W group (*p* = 0.033).

Theta band power increased in L at Pz during gaming, and was not significantly different between groups (Figure 10A). During gaming, power in beta 1 decreased frontocentrally in L and increased parietooccipitally in W (only the difference at Pz remained significant after FDR correction). Beta 2 was not significantly different between groups or conditions, after applying correction for multiple comparisons. Based on the spatial distribution of gaming alpha (posterior increase in W, widespread decrease in L), beta 1 and theta (lack of modulation in W, posterior increase in L) frequencies, it appears that W were more relaxed and detached, while L were stressed or aroused (Nowak and Marczynski, 1981; Ray and Cole, 1985; Niedermeyer and Lopes da Silva, 2005).

#### Intra Brain PLV

**Figure 11:**
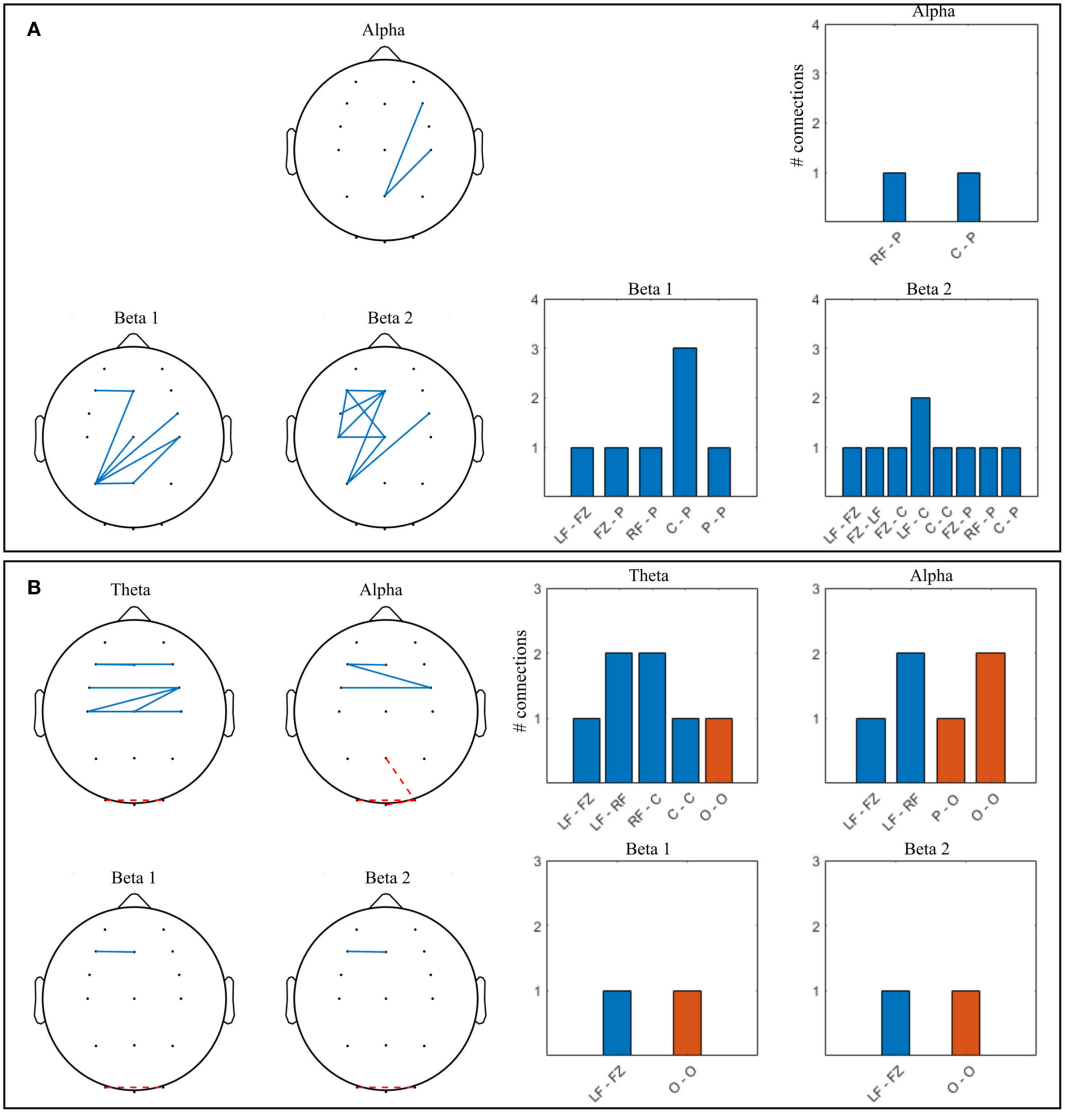
Significant connections in competitive gaming vs. baseline grand average (*p* <0.01, FDR corrected); theta = 4–7 Hz, alpha = 8–12 Hz, beta 1 = 13–20 Hz, beta 2 = 21–30 Hz. **(A)** winners (W), **(B)** losers (L). Grouped locations: LF, left frontal; RF, right frontal; C, central; P, parietal; O, occipital and topographical plots. Blue, continuous, increase from baseline; orange, dashed, decrease from baseline.

Both W and L groups showed significant increase in connectivity during gaming as compared to baseline in the alpha, beta 1 and beta 2 bands. Theta band PLV increased significantly in L but did not change in W. Significant connections for W and L groups are shown in Figures 11A,B respectively, as resulted from a two-tailed bootstrap test of gaming vs. baseline (*p* <0.01, FDR corrected).

There was a significant increase in alpha connectivity between Pz (location of modulation) and right frontocentral area in W group (*p* = 0.002), and a significant decrease between Pz and right occipital area in L group (*p* = 0.001). In addition, alpha connectivity increased between frontal and frontocentral locations in L. There is a higher density of significant changes in connectivity during gaming in W group compared to L, in beta 1 and beta 2 frequency bands. Connectivity in the theta band increases in L during gaming within frontal and central areas. W increase beta 1 and beta 2 PLV between frontocentral and parietal areas, and beta 2 within the left frontocentral hemisphere. L changes in connectivity mostly occur frontocentrally, across hemispheres, with midline to left frontal increase and left to right occipital decrease present in all frequency bands.

In summary, both spectral and connectivity analysis indicate that W and L modulation occurs in different regions of the cortex. W increased power in central, parietal and occipital areas, while connectivity strengthened mainly centroparietally. In L, connectivity increase occurred frontocentrally, between hemispheres. Midline to left frontal connectivity increased in both beta bands in W and in all bands in L.

#### Interbrain PLV

**Figure 12:**
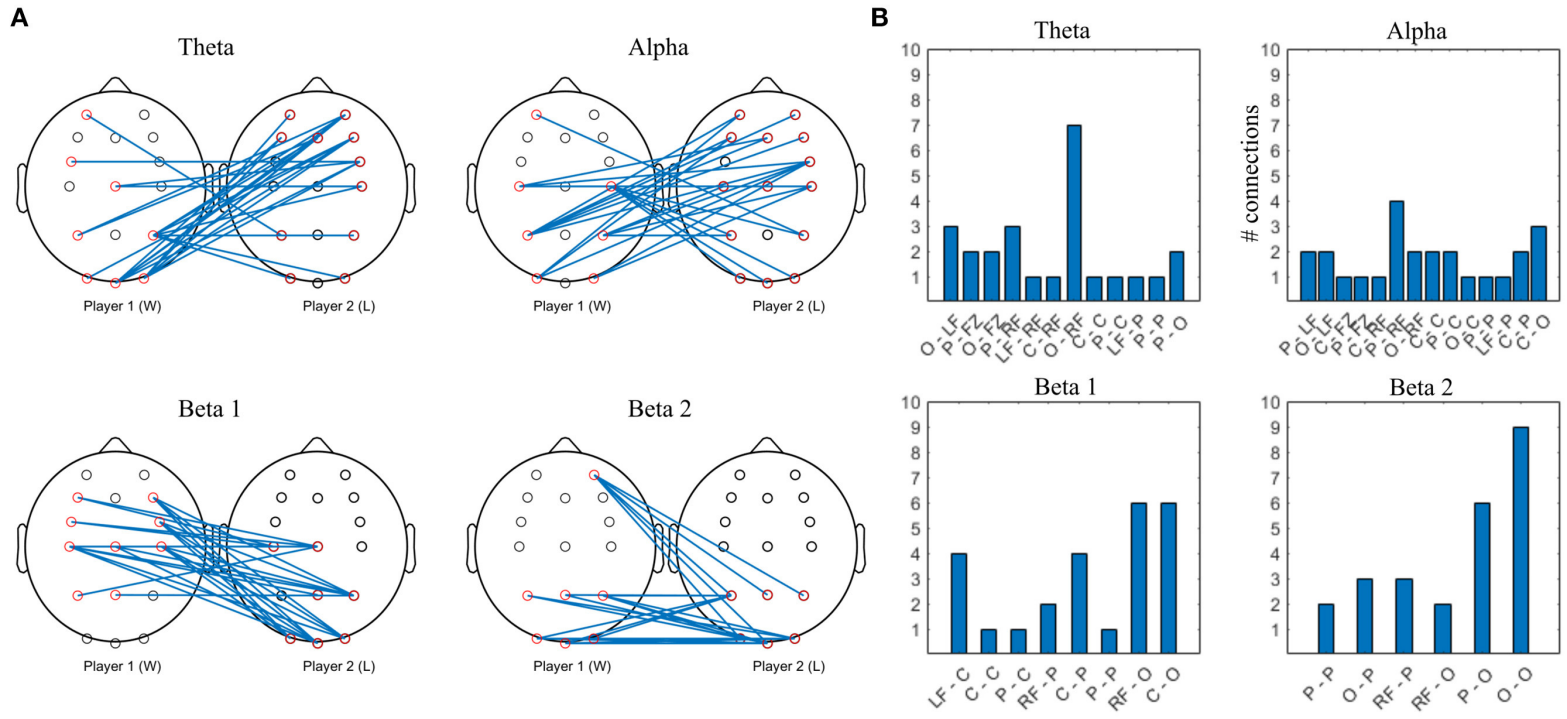
Top 10% strongest connections in competitive gaming, interbrain connectivity. **(A)** topographical plot. **(B)** grouped locations. W, first letter; L, second letter. LF, left frontal; RF, right frontal; C, central; P, parietal; O, occipital.

Permutation testing of interbrain connectivity in the competitive mode indicate that there is no significant synchrony during baseline, and that all values of grand average gaming intersynchrony are significant (*p* <1 ×10^−12^). The strongest 10% connections are shown in Figure 12 for all frequency bands, and violin plots showing individual pairs' interconnectivity values are presented in the Supplementary Figure S7.

Theta band interconnectivity is mainly asymmetrical, between W parietooccipital and L frontocentral, with the exception of symmetrical central-central intersynchrony. Overall, the cortex of the L is more involved compared to W, with both anterior and posterior connections bilaterally. Alpha band hypersychrony is dominated by parietal and occipital to frontal interconnections between W and L. There is less involvement of W players' midline locations compared to L. Patterns of the strongest interconnections in the beta 1 and beta 2 bands appear to be mirrored, particularly in the posterior areas.

### Gaming Workload

The perceived experimental workload was measured by NASA TLX questionnaires in both gaming paradigms. Friedman's test (*p* <0.05) showed no statistically significant difference in any group or gaming paradigm. Likewise, no significant difference was found between groups for any category (Wilcoxon signed rank test, *p* <0.05). In collaborative gaming, the highest scores were achieved in *Performance*, followed by *Effort* and *Mental* in group D, whereas in group ND the generally higher scores were achieved in *Mental*, followed by *Effort* and *Performance* categories. In competitive gaming, the largest score was achieved for the category *Effort* in both groups, with W and L experiencing a similar level of task load.

## Discussion

The gaming paradigms presented in this study consisted of modulation of relative alpha band power from the midline parietal location, Pz, during multi-user neurofeedback collaboration or competition.

Although intra brain connectivity and hyperconnectivity have been studied with various BCI game paradigms, the novelty of this study is that it investigates the effect of multiuser neurofeedback gaming on functional connectivity, offering new insight into the social interaction and strategies of users playing in competition or collaboration. The game presented players with a two faceted challenge: they had to learn brain activity modulation, and to adapt it so as to meet a threshold enforced externally, by the co-player. By using the same task in different social paradigms, we hoped to discern characteristic neural patterns of the two mental strategies, and not only for the particular task of NF.

Firstly, we aimed to identify patterns of neural activity and connectivity in multiuser BCI. Secondly, we investigated how players' neural characteristics relate to the collaborative or competitive game outcome. Thirdly, this study aimed to characterize the effects of competition and collaboration in neurofeedback on the dyad's neural interaction.

We found that NF during both collaboration and competition modulate players' spectral power and brain synchrony with respect to resting state, and that the patterns of connectivity differ considerably between the two paradigms. Collaborative players with higher resting state RA modulated their brain oscillations more readily compared to their pairs'. In competition, the winners displayed neural signatures indicative of relaxation, while the opposite was true for the losers. Interbrain synchrony, found during both gaming paradigms, also differed between collaboration and competition. Results of the former suggest planning and social interaction occurred to a similar extent in the team, while in the latter, losers' approach was associated with monitoring others' progress and strategizing.

### Power Spectrum

We analyzed changes in the relative alpha power at Pz, as this was a control signal for NF, followed by the analysis of absolute power in four frequency bands during baseline and NF.

Higher content of baseline alpha has been identified as a predictor of alpha neurofeedback success (Wan et al., 2014), while increased alpha power content during a task is known to be related to better allocation of cognitive resources (Klimesch et al., 2007). In this study, a higher content of RA power in D group in the collaborative game, as well as higher content of the absolute alpha at Pz, were indicative of a better ability to modulate the absolute alpha power during gaming, to adapt to the ND player. This is in line with findings of Wan et al. (2014) showing that higher RA at the NF site is a predictor of better self-regulation of brain activity. Both D and ND players decreased their alpha power during the game, and all collaborating dyads downregulated RA. Although the sample size of this study was relatively small (10 dyads), increasing the number of participants would not necessarily affect our conclusions, since pairs are likely to choose the easier option of decreasing RA, unless otherwise instructed.

The resting state RA content at the NF site was not a consistent predictor of success of the competitive game. While W group's baseline RA was higher at Pz than that of L, the difference did not remain significant after FDR correction. The only difference between groups' absolute alpha was that W had significantly higher power occipitally than L. Significant theta activity changes during gaming as compared to baseline were seen in L and D groups. Based on PSD topography of competing dyads, it seems that W won due to their ability to detach, by maintaining RA at baseline level, as well as because L decreased RA during the game. From literature on the relation between theta activity and emotional processing (Yun et al., 2012; Balconi et al., 2015; Balconi and Vanutelli, 2018), W group might have also been less involved emotionally in the game than the latter. However, these remain speculations, as NASA-TLX results did not indicate significant differences between the groups. The hypothesis could be verified by administering more specific questionnaires such as the Self-Assessment Manikin (Bradley and Lang, 1994), or assessing the flow related to the task (Jackson and Marsh, 1996).

Frontal theta, generally associated with higher mental workload and planning (Cavanagh and Frank, 2014; Domic-Siede et al., 2019), was not modulated in any of the four groups, indicating that the game was not cognitively demanding. This is substantiated by the NASA-TLX findings–there were no significant differences in perceived effort or mental load between groups (D, ND and W, L). In addition, there was no significant change across sessions in any of the TLX domains.

Spectral power modulation was similar in collaborative D and competitive L groups in the alpha (decrease) and beta 1 (frontal decrease) bands, indicating that they might have used a similar approach. In the case of the former, power modulation led to the team's success, while the latter performed poorly in the game.

Taken together, results from competitive and collaborative games show that the context of the game is as relevant as the participants' ability to self-regulate their brain activity. It also shows that playing against a dynamic, externally driven target does not necessarily modulate EEG power in a predefined direction, as this is not an explicit rule of the game. This is in contrast to single player NF where the target is set with respect to one's own EEG. Thus, modulating EEG power in a specific direction in a multiuser NF with an externally driven target would require an additional set of rules.

### Intrabrain Connectivity

Collaborative and competitive gaming led to significant changes in connectivity between brain regions with respect to resting state. The most striking difference is the considerably smaller connectivity changes in the competitive as compared to the collaborative task. Modulation of PLV also differed between two players in a dyad, in particular in competitive gaming.

Among all four groups (D, ND, W and L), D players had the strongest long and short-range increase in connectivity in both beta bands, while ND increased long-range connectivity, potentially between default mode network (DMN) structures (Raichle et al., 2001) in the theta band. Connectivity patterns of the two groups in competition (W and L) were also markedly dissimilar, revealing the relationship between the task (and performance) and functional integration of brain regions. W displayed long range change in connectivity in alpha and both beta bands while L had short range interhemispheric frontal changes in PLV in all frequency bands. Notably, winners of competitive gaming are the only players who did not modulate theta connectivity.

Theta band frontocentral to parietal connections, present in both D and ND during collaboration, are indicative of internally directed attention, as highlighted by Kam et al. (2019), whose study revealed an interaction between DMN structures and the frontoparietal network in the 4–7 Hz band, but not in the alpha band. Moreover, Fareri et al. (2020) found an interaction between DMN and attentional/control networks during activities with positive social outcome (i.e., reciprocation vs. violation of trust), that might be present in collaborative gaming. Increase in theta synchrony in both groups might thus reflect the collaborative nature of social interaction.

Frontal and frontocentral functional connectivity increase in alpha (L) and theta (D, L groups) suggest integration of the areas across hemispheres (Varela et al., 2001; Valencia and Froese, 2020). Thus, although theta power was not modulated frontally, synchrony of brain areas can be related to cognitive control and reinforcement learning (Cavanagh et al., 2010; van Driel et al., 2015). This is in contrast to W group, where theta band synchrony showed no change, substantiating the assumption that W were able to detach from the game more than L (Balconi and Vanutelli, 2018).

Dominant players seemed to work the hardest of all four groups, with power decrease in all bands but theta, and a high density of beta connectivity increase. A better ability for network reconfiguration, particularly long-range, is thought to be related to improved attention and cognitive task performance (Rogala et al., 2020). Beta connectivity increase allows long-range communication in the cortex (Siegel et al., 2012), while also being associated with movement and motor imagery, somatosensory integration and increased cognitive load (Betti et al., 2021).

On the one hand, connectivity results in the alpha and theta bands during collaboration indicate that (i) decrease in alpha power is not necessarily linked to significant changes in alpha connectivity (D group), and (ii) players who were less proactive in modulating alpha (ND group) increased connectivity between structures of the control network and DMN. On the other hand, competition accentuated the superiority of players who were able to separate from the NF game and their opponents, in contrast to those who seemed to employ more resources and potentially work harder. Together, these might indicate that W learned over the first two training sessions that relaxing and maintaining RA is the winning strategy, while L used an inappropriate mental strategy that decreased alpha power.

It should be noted that these observations are valid for this particular NF strategy. If the game was based on e.g., upregulation of frontal theta or beta band power, successful players would apply different mental strategies and that would result in different intra and interbrain connectivity. Thus, definition of “successful” strategy is highly dependent on the rules of the game.

### Interbrain Connectivity

Dikker et al. (2021) proposed a matrix of sources accounting for inter-brain coupling, outside of that induced by the experimental paradigm. To isolate the effects of two-player BCI gaming on brain hypersynchrony, we attempted to minimize these factors. The impact of social behavior was minimal as dyads were not closely related and were not allowed to communicate (verbally or non-verbally) during the experiment. They were facing the same direction and did not make eye contact while gaming. Exogenous stimuli (shared environment) leading to brain synchrony affected all pairs of collaborative and competitive gaming. Personality traits and mental states of players were neither controlled for, nor accounted for. However, cognitive load assessment (NASA TLX) did not show significant differences between groups in either task. A factor of interest in multiplayer BCI gaming is extrinsic motivation—is the task engaging enough to increase inter-brain coupling? We showed that gaming interconnectivity is significant and could not arise by chance, which is not the case for resting state connectivity. Thus, we believe that the effects presented in this study are due in great part to the task of engaging in a multiplayer neurofeedback-based game.

Collaborative and competitive gaming give rise to markedly different patterns of the strongest interbrain links, particularly in the alpha and theta bands. No interbrain connectivity was found during baseline, indicating that multiuser gaming, rather than shared environment, gives rise to functional brain integration.

Collaboration is linked to theta intersychrony of frontocentral regions of both groups (D, ND), and mostly symmetrical posterior alpha interconnectivity, with higher involvement of right frontocentral locations in ND players. A coordination counting task showed similar involvement of central, parietal and occipital areas in alpha interbrain synchrony, especially in female dyads (Mu et al., 2016), which the authors relate to dyads' intent to coordinate. A study of hyperconnectivity during a cooperative decision task has reported theta synchrony in the central-frontal area and alpha synchrony in centroparietal regions, in line with the findings of our study (Hu et al., 2018), while near-infrared hyperscanning of a cooperative cognitive task also reported higher synchrony of the dyads' PFC after feedback (Balconi and Vanutelli, 2017).

In contrast to collaboration, the pattern of interbrain connectivity during competition is more asymmetrical in theta and alpha frequency bands. W display lateralization of the frontocentral cortex theta and alpha interconnections, as well as occipital (W) to frontal interconnections. L group's anteriofrontal regions in theta and alpha interconnectivity, areas linked to social participation and predicting others' intentions (Toppi et al., 2016), are more connected to their competitors' cortex than vice-versa.

In collaborative gaming, the interaction in the theta band is more symmetrical prefrontally, suggesting that collaboration is linked to social exchange and strategic planning in both players, while competing determines the loser, but not the winner, to mentalise. The PFC is involved in social interaction and individuals partaking in competitive tasks reportedly display medial PFC activation linked to separation from their competitors (Decety et al., 2004), as well as perception and prediction of others' intentions (Cui et al., 2012; Hu et al., 2018). The medial PFC role in competition is to coordinate emotional and cognitive neural activity in the whole cortex, since the competitive mindset requires social comparison—following one's actions alongside those of the competitor (Decety et al., 2004).

Together with results of theta intrabrain connectivity, it appears that the competitive game determined L players to follow their opponents' behavior or use metalizing strategies. This did not help them win the NF-based game, which required players to detach from the game in order to increase alpha, rather than strategies. As such, although L did not perform well in competitive NF games, they might have the upper hand in a different multiuser BCI gaming paradigm like motor imagery ping pong (Brain Arena), (Bonnet et al., 2013) or games controlled by somatosensory evoked potential (Mind the Sheep!), (Gürkök et al., 2013).

In the alpha band, intersynchrony between anteriofrontal locations (PFC) of the L and centroparietal areas of the W are similar to the pattern found in a study investigating interbrain synchronization (PLV) between speakers and listeners after accounting for the brain audio envelope, which the authors attribute to dyad's brain to brain entrainment (Pérez et al., 2017). The interconnections reported in the lower beta band (15–20 Hz) also show similarity to our results, and an analogy between W-speaker and L-listener: mainly frontal involvement of the speaker's cortex and increased midline interconnectivity in both speaker and listener (Pérez et al., 2017). These could indicate that the losers were more successful at monitoring and anticipating the other's progress, in a similar fashion to listeners following speech. This supports the assumption that L used a gaming strategy that would have been successful in a different type of game design, with higher emphasis on social communication outside of linguistic communication.

Collaboration, but not competition is associated with alpha band interbrain connectivity between the location of neurofeedback (Pz), potentially indicating functional integration of the brain regions used to control the game (Valencia and Froese, 2020). Interconnectivity results indicate that competition is linked to a wider, less focalized cortical involvement compared to collaboration.

Asymmetrical patterns dominating in the higher bands may be related to interparticipant top-down modulation (Dumas et al., 2010) and the existence of a leader. Planning and attentional processes are mediated by beta rhythms presumably originating in higher cognitive locations such as the PFC, and terminating in the sensory or posterior cortex (Lee et al., 2013). In our study, high beta band connections between frontal and anteriofrontal locations in L and D groups, and parietooccipital of W and ND are possibly linked to the formers' attempt to mentalise. Overall, this might indicate that interaction during two-player gaming is linked to well-defined roles, irrespective of the gaming mode, but this requires further investigation.

In conclusion, collaborative gaming determined the non-dominant participant to be less active, and the dominant player to employ strategies that lower their relative alpha and earn points for the team. We found neural indices of collaboration in both spectral and connectivity analysis. Competitive neurofeedback was won by the players who managed to maintain a similar level of relative alpha to that of the resting state, aided by their opponents downregulating relative alpha power. Losers appeared to use the inappropriate mental strategies for this type of game. While personality traits and mental states are important for a multiuser interaction (Wood and Kober, 2018; Reinero et al., 2021), game design is equally important. A game in the current study was designed to allow simple adaptation to competitive or collaborative mode while keeping the same GUI and was limited to basic graphical representation to exclude the influence of a complex interface on the interpretation of results. It is however possible that a more engaging and entertaining game would result in increased motivation and improved performance, leading to different brain activity modulation in the theta and beta bands.

## Limitations of The Study

This study aimed to characterize the effect of EEG neurofeedback-based multiuser gaming in competitive and collaborative modes on neural features and brain functional connectivity. One of the main shortcomings of the study is related to the experimental setup—the common interface and shared environment for both players in a dyad, constitute confounding variables and may give rise to spurious connectivity. Moreover, PLV is affected by volume conduction and, due to the low number of sensors used in this study, we were unable to reduce these effects through source analysis. However, the two-stage statistical validation using permutation-based methods minimized the interbrain connectivity arising from players sharing the room. In addition, the same study setup was used for both competitive and collaborative gaming. As such, differences in strategy between the two paradigms should not be influenced by the setup. Intrabrain connectivity was analyzed using the difference between gaming and baseline, to isolate effects of the tasks as much as possible.

The current work used a relatively small sample size to study collaboration and competition (*N* = 10 dyads for each), and we could not ascertain the ability of players to compete or collaborate based on their resting state brain signatures. However, results indicate a tendency for players displaying higher resting state alpha to work harder during collaborative gaming, which could also be caused by the difficulty of upregulating alpha. To further investigate this, participants should undergo a few sessions of individual neuromodulation practice before playing the multiuser game. We believe that, without further instructions (i.e., increase RA), the approach of the collaborating dyads to lower RA would be consistent across pairs, irrespective of the sample size.

As previously mentioned, a suitable game strategy is highly dependent on the NF paradigm and on the partner's performance. Thus, dominant or winning players might be losers or non-dominant and might have applied different mental strategy if paired differently. A study where all players would play against several randomly chosen players out of remaining 19 participants (10 couples) might show how much individual's gaming strategy and brain connectivity depend on the gaming strategy of their partner in either collaborative or competitive game.

## Future Steps

Future studies should employ single-player controls as a direct comparison of the effect of competing/collaborating interaction during neurofeedback. Real-time adaptation of game difficulty could be implemented in order to increase players' engagement and even out the perceived task parameters (effort, workload) across participants (Stevens et al., 2010; Darzi and Novak, 2021). A direct comparison between neurofeedback-based multiplayer games and other brain-controlled games (MI, SSEP) would determine characteristics of player performance. Two aspects of the collaborative task could be modified in the future to provide a better picture of the paradigm's effects. Firstly, a higher sample size should be used to determine if the collaborating dyads' strategy to decrease RA remained consistent across pairs. Secondly, more specific instructions (i.e., increase, rather than modulate, RA) would condition a different approach to the game.

## Data Availability Statement

The raw data supporting the conclusions of this article will be made available by the authors, without undue reservation.

## Ethics Statement

The studies involving human participants were reviewed and approved by College of Science and Engineering Ethics Committee, University of Glasgow. The patients/participants provided their written informed consent to participate in this study.

## Author Contributions

IS substantially contributed to data analysis and manuscript writing. FP substantially contributed to the experimental design, collected the data, and revised the manuscript. HD contributed to study design and revised the manuscript. AV conceptualized the study, substantially contributed to data interpretation, and manuscript writing. All authors contributed to the article and approved the submitted version.

## Funding

This work has been supported by Indonesian Endowment Fund for Education Ph.D. scholarship and RCUK EPSRC Ph.D. scholarship EP/R513222/1ENG.

## Conflict of Interest

The authors declare that the research was conducted in the absence of any commercial or financial relationships that could be construed as a potential conflict of interest.

## Publisher's Note

All claims expressed in this article are solely those of the authors and do not necessarily represent those of their affiliated organizations, or those of the publisher, the editors and the reviewers. Any product that may be evaluated in this article, or claim that may be made by its manufacturer, is not guaranteed or endorsed by the publisher.
