# Supplementary material for: Brain Synchrony in Competition and Collaboration During Multiuser Neurofeedback-Based Gaming
Source: Front Neuroergon. 2021 Nov 1;2:749009. doi: 10.3389/fnrgo.2021.749009 (PMC10790838; doi:10.3389/fnrgo.2021.749009)
Supplement: Supplementary file 1 [file Image_1.pdf]

Supplementary material

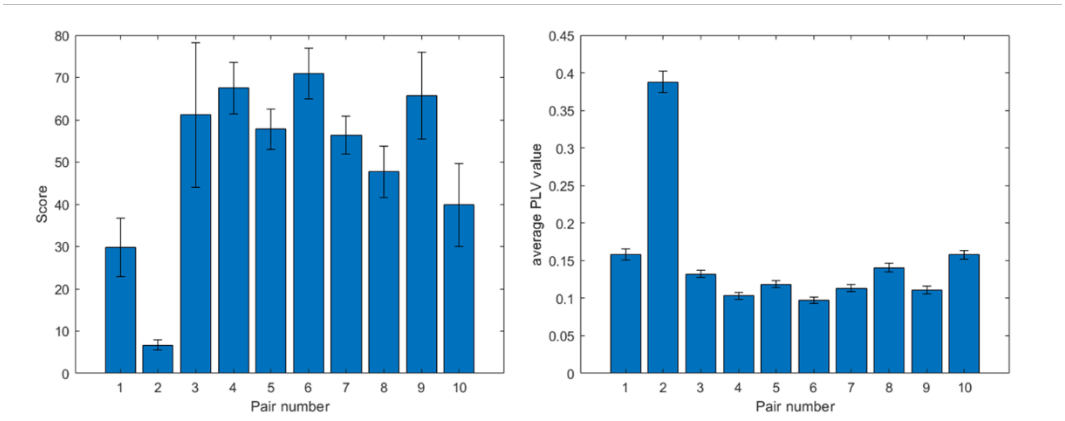

Figure S 1 - Average sub-session score count (left) and average PLV value in alpha band (right) for each pair in collaborative gaming.

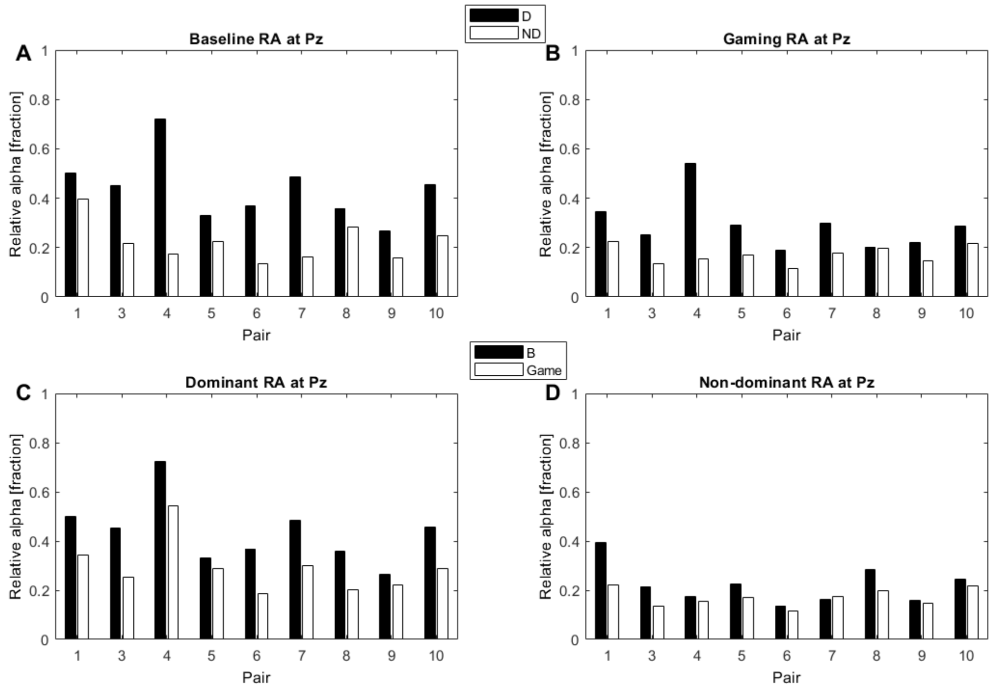

Figure S 2 - - Bar plot of relative alpha power at Pz; A – baseline D (black) and ND (white); B – gaming D and ND; C – D group during baseline (black) and gaming (white); D – ND group during baseline and gaming. Pair 2 excluded.

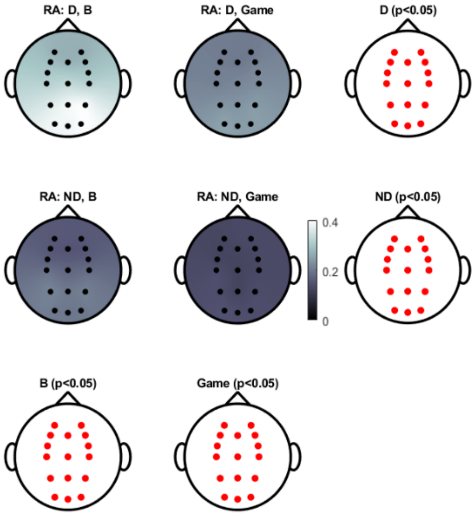

Figure S 3 - Relative power during baseline and collaborative gaming for D and ND groups, in alpha band. Significant differences from permutation testing shown in red (t-test, p<0.05, FDR corrected).

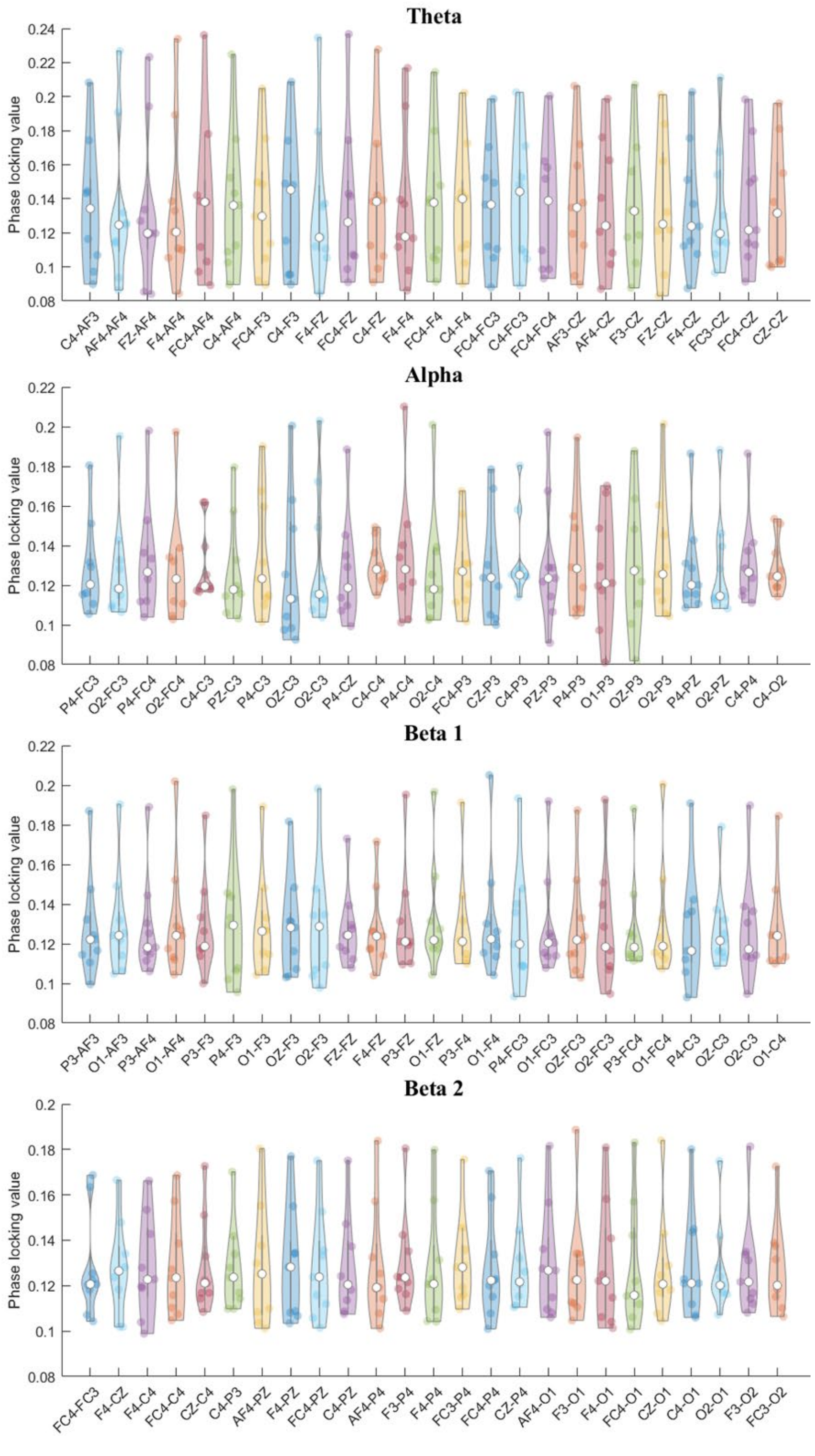

Figure S 4 - Violin plots of the strongest 10% interbrain connections (electrode locations) during collaborative gaming in all frequency bands, with connectivity values from individual pairs shown (excluding Pair 2).

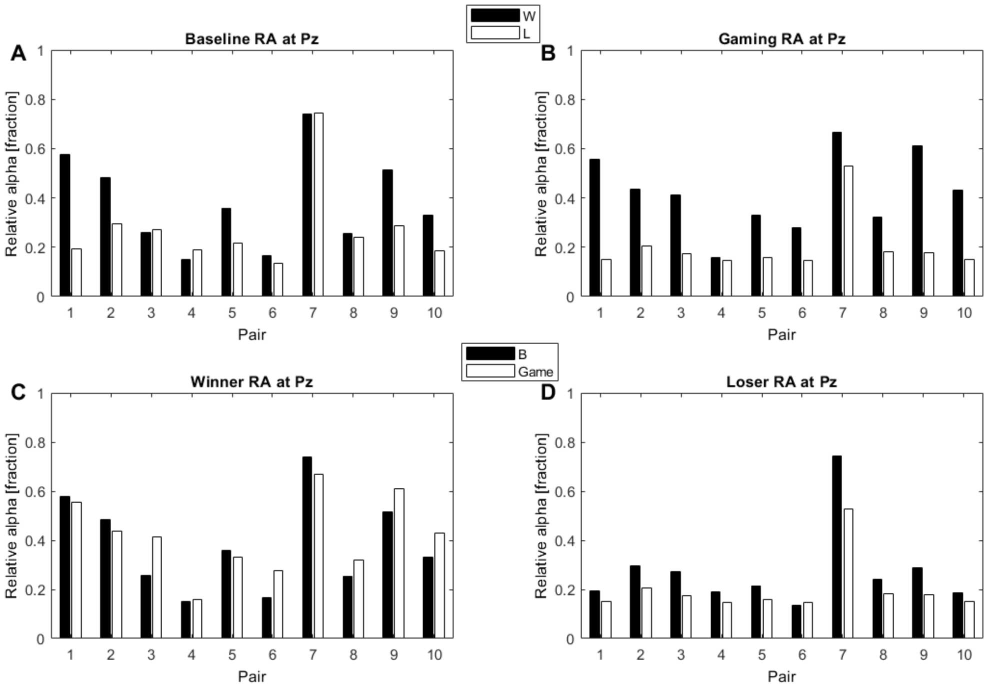

Figure S 5 - Bar plot of relative alpha power at Pz; A – baseline W (black) and L (white); B – gaming W and L; C – W group during baseline (black) and gaming (white); D – L group during baseline and gaming.

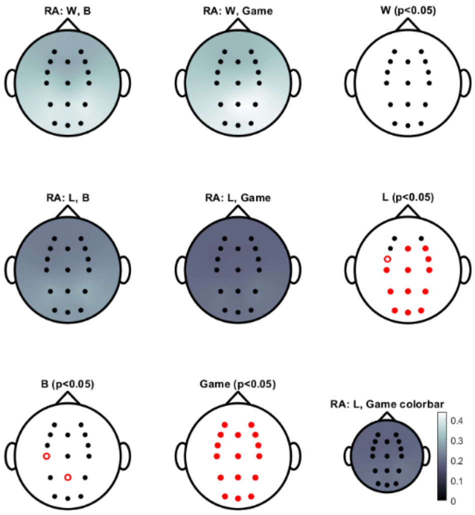

Figure S 6 - Relative power during baseline and competitive gaming for W and L groups, in alpha band. Significant differences from permutation testing shown in red (t-test, p<0.05, FDR corrected).

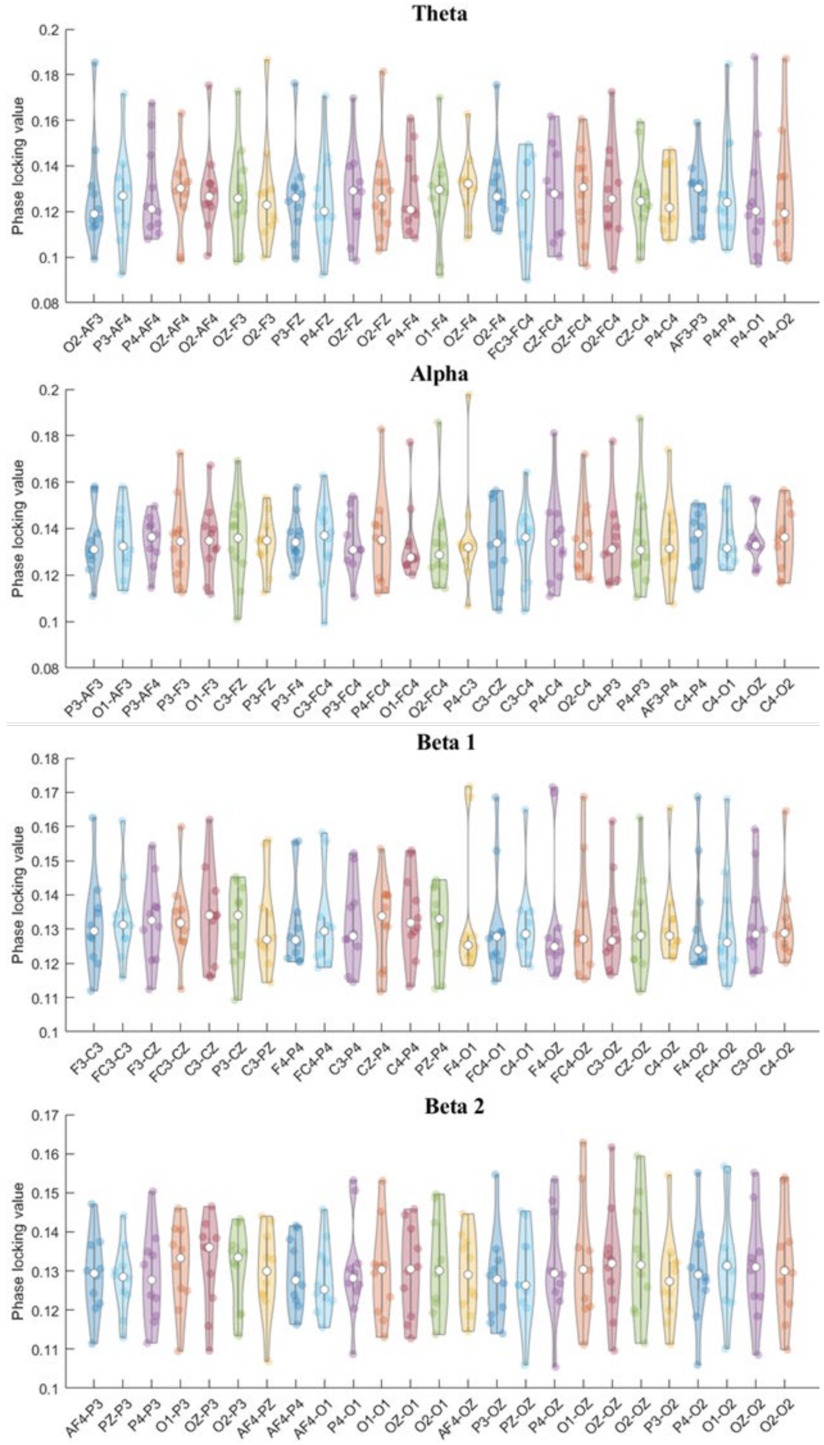

Figure S 7 - Violin plots of the strongest 10% interbrain connections (electrode locations) during competitive gaming in all frequency bands, with connectivity values from individual pairs shown.
